# Supplementary material for: Synergistic Effect of Acetazolamide-(2-hydroxy)propyl β-Cyclodextrin in Timolol Liposomes for Decreasing and Prolonging Intraocular Pressure Levels
Source: Pharmaceutics. 2021 Nov 25;13(12):2010. doi: 10.3390/pharmaceutics13122010 (PMC8709067; doi:10.3390/pharmaceutics13122010)
Supplement: Supplementary file 1 [file pharmaceutics-13-02010-s001.zip › pharmaceutics-1408756-supplementary.pdf]

pharmaceutics MDPI

Supplementary Materials: Synergistic effect of acetazolamide-hydroxypropylβcyclodextrin in timolol liposomes for decreasing and prolonging the intra-ocular pressure levels

Carmen M. Arroyo-García, Daniela Quinteros, Santiago D. Palma, Cesáreo J. Jiménez de los Santos, José R. Moyano, Antonio M. Rabasco and María Luisa González-Rodríguez

Table S1. Stability study realized for three months at 4 °C. Mean ± SD.

	0 months	1 month				3 months
		7 days	14 days	21 days	30 days	
**d_H (nm)**	**200.6 ± 1.6**	198.6 ± 2.10	197.1 ± 1.93	194.6 ± 2.72	193.2 ± 1.95	194.3 ± 2.52
**PdI**	0.13 ± 0.04	0.15 ± 0.03	0.16 ± 0.04	0.14 ± 0.02	0.17 ± 0.03	0.17 ± 0.02
**ζ (mV)**	19.5 ± 0.3	19.9 ± 0.4	19.6 ± 0.3	20.1 ± 0.3	20.1 ± 0.4	21.1 ± 0.2
**EE TM (%)**	82.1 ± 1.32	82.0 ± 1.37	82.1 ± 1.41	81.8 ± 2.34	80.1 ± 2.01	80.4 ± 1.78
**EE Acz (%)**	72.6 ± 1.49	72.3 ± 1.51	71.5 ± 2.12	70.6 ± 1.98	70.0 ± 1.37	69.6 ± 1.21

Table S2. Mean values of HPβCD concentrations and acetazolamide (Acz) concentration dissolved in the presence of Acz/HPβCD, Acz/HPβCD/Ch or Acz/HPβCD/Chems. These mean values (S_t) and their SD are plotted in Figure 1a. Bold values represents S_0. Log (S_t-S_0) mean values and their SD are also tabulated for each formulation and they are plotted in Figure 1b.

HPβCD	Acz/HPβCD	Acz/HPβCD/Ch	Acz/HPβCD/Chems	Log(S_t–S_0) Acz/HPβCD	Log(S_t–S_0) Acz/HPβCD/Ch	Log(S_t–S_0) Acz/HPβCD/Chems
0	**3.48 ± 0.18**	**2.57 ± 0.15**	**2.94 ± 0.15**	-	-	-
20	6.80 ± 0.30	4.78 ± 0.13	6.76 ± 0.12	0.53 ± 0.02	0.34 ± 0.02	0.58 ± 0.01
40	9.30 ± 0.12	6.52 ± 0.12	9.81 ± 0.21	0.77 ± 0.01	0.60 ± 0.01	0.84 ± 0.00
60	13.30 ± 0.46	8.52 ± 0.20	13.04 ± 0.17	1.00 ± 0.02	0.77 ± 0.00	1.00 ± 0.01
80	16.30 ± 0.70	11.10 ± 0.26	15.25 ± 0.22	1.11 ± 0.02	0.93 ± 0.01	1.09 ± 0.01
100	19.40 ± 0.10	13.70 ± 0.72	18.89 ± 0.19	1.20 ± 0.01	1.05 ± 0.02	1.20 ± 0.00
